# Cardiovascular magnetic resonance accurately detects obstructive coronary artery disease in suspected non-ST elevation myocardial infarction: a sub-analysis of the CARMENTA Trial

**DOI:** 10.1186/s12968-021-00723-6

**Published:** 2021-03-22

**Authors:** Yvonne J. M. van Cauteren, Martijn W. Smulders, Ralph A. L. J. Theunissen, Suzanne C. Gerretsen, Bouke P. Adriaans, Geertruida P. Bijvoet, Alma M. A. Mingels, Sander M. J. van Kuijk, Simon Schalla, Harry J. G. M. Crijns, Raymond J. Kim, Joachim E. Wildberger, Jordi Heijman, Sebastiaan C. A. M. Bekkers

**Affiliations:** 1 Department of Radiology and Nuclear Medicine, Maastricht UMC+, Maastricht, The Netherlands; 2 Department of Cardiology, Maastricht UMC+, Maastricht, The Netherlands; 3 Cardiovascular Research Institute Maastricht (CARIM), Maastricht University, Maastricht, The Netherlands; 4 Department of Clinical Chemistry, Maastricht UMC+, Maastricht, The Netherlands; 5 Department of Clinical Epidemiology & Medical Technology Assessment (KEMTA), Maastricht UMC+, Maastricht, The Netherlands; 6 Duke Cardiovascular Magnetic Resonance Center, Duke University Medical Center, Durham, NC USA; 7 Maastricht University Medical Center, P. Debyelaan 25, P.O. Box 5800, 6202 AZ Maastricht, The Netherlands

**Keywords:** Non-ST elevation myocardial infarction, Coronary artery disease, Cardiovascular magnetic resonance, Coronary angiography, Diagnostic accuracy, High-sensitivity cardiac troponin, Acute coronary syndrome

## Abstract

**Background:**

Invasive coronary angiography (ICA) is still the reference test in suspected non-ST elevation myocardial infarction (NSTEMI), although a substantial number of patients do not have obstructive coronary artery disease (CAD). Early cardiovascular magnetic resonance (CMR) may be a useful gatekeeper for ICA in this setting. The main objective was to investigate the accuracy of CMR to detect obstructive CAD in NSTEMI.

**Methods:**

This study is a sub-analysis of a randomized controlled trial investigating whether a non-invasive imaging-first strategy safely reduced the number of ICA compared to routine clinical care in suspected NSTEMI (acute chest pain, non-diagnostic electrocardiogram, high sensitivity troponin T > 14 ng/L), and included 51 patients who underwent CMR prior to ICA. A stepwise approach was used to assess the diagnostic accuracy of CMR to detect (1) obstructive CAD (diameter stenosis ≥ 70% by ICA) and (2) an adjudicated final diagnosis of acute coronary syndrome (ACS). First, in all patients the combination of cine, T2-weighted and late gadolinium enhancement (LGE) imaging was evaluated for the presence of abnormalities consistent with a coronary etiology in any sequence. Hereafter and only when the scan was normal or equivocal, adenosine stress-perfusion CMR was added.

**Results:**

Of 51 patients included (63 ± 10 years, 51% male), 34 (67%) had obstructive CAD by ICA. The sensitivity, specificity and overall accuracy of the first step to diagnose obstructive CAD were 79%, 71% and 77%, respectively. Additional vasodilator stress-perfusion CMR was performed in 19 patients and combined with step one resulted in an overall sensitivity of 97%, specificity of 65% and accuracy of 86%. Of the remaining 17 patients with non-obstructive CAD, 4 (24%) had evidence for a myocardial infarction on LGE, explaining the modest specificity. The sensitivity, specificity and overall accuracy to diagnose ACS (n = 43) were 88%, 88% and 88%, respectively.

**Conclusion:**

CMR accurately detects obstructive CAD and ACS in suspected NSTEMI. Non-obstructive CAD is common with CMR still identifying an infarction in almost one-quarter of patients. CMR should be considered as an early diagnostic approach in suspected NSTEMI.

*Trial registration*. The CARMENTA trial has been registered at ClinicalTrials.gov with identifier NCT01559467.

## Background

Non-ST elevation myocardial infarction (NSTEMI) is an important acute manifestation of coronary artery disease (CAD) and a leading cause of death [1]. Early invasive coronary angiography (ICA) to assess the presence and severity of CAD is recommended in patients at high risk of adverse events. However, visual assessment is prone to under- or overestimate stenosis severity [2] and it is difficult to angiographically identify the culprit or ischemia-causing lesion [3].

High-sensitivity cardiac troponin (hs-cTn) assays have very high sensitivity for myocardial injury, but their specificity to detect myocardial infarction (MI) is limited [1]. This may partly explain why up to 30% of patients with suspected NSTEMI and elevated hs-cTn levels do not have obstructive CAD [4]. Nonetheless, MI with non-obstructive coronary arteries (MINOCA) is not uncommon. Potential etiologies include plaque rupture with distal embolization and/or temporary coronary artery occlusion with spontaneous reperfusion, as well as non-coronary etiologies including myocarditis, (stress) cardiomyopathy, structural and hypertensive heart disease, and pulmonary embolism. Altogether, these data raise the question whether ICA is still appropriate as an initial diagnostic test in patients with low-to-intermediate risk suspected NSTEMI.

The strength of cardiovascular magnetic resonance (CMR) is its ability to assess regional and global myocardial function, presence and pattern of scar, myocardial ischemia and to differentiate between coronary and non-coronary etiologies in a single investigation [5]. Vasodilator stress-perfusion CMR accurately detects obstructive CAD in a stable chest-pain population [6], and a CMR-guided strategy lowers the probability of inappropriate ICA [7, 8]. However, so far, no study has evaluated the diagnostic value of CMR in patients with suspected NSTEMI in the current hs-cTn era. The purpose of this study was to investigate the diagnostic value of CMR prior to ICA in this patient population. We evaluated both the accuracy of CMR in detecting obstructive CAD on ICA, as well as for detecting a final adjudicated diagnosis of acute coronary syndrome (ACS), given the prevalence of MINOCA.

## Methods

### Study population

This is a sub-analysis of the CARMENTA trial, a single-center randomized controlled trial [9]. In brief, CARMENTA included 207 consecutive patients without a known history of CAD or cardiomyopathy who were admitted because of acute chest pain, normal or non-diagnostic electrocardiogram and high sensitivity cardiac troponin T (hs-cTnT) values > 14 ng/L, at baseline or 3 h after presentation (i.e., suspected NSTEMI). Patients were randomized to routine clinical care, a computed tomography angiography (CTA)-first strategy or a CMR-first strategy. A recommendation based on the CTA or CMR results was given to the treating cardiologist. However, follow-up diagnostic testing, medical therapy, and discharge were left at the discretion of the treating cardiologist. For the present sub-analysis, only patients from the CMR-first strategy who underwent CMR and subsequent ICA were included.

### CMR scan protocol

The protocol included scout, anatomical, cine and T2-weighted images, rest- and adenosine stress-perfusion, and late gadolinium enhancement (LGE)-CMR. Because stress testing has historically been considered inappropriate for NSTEMI patients, a stepwise approach was used in CARMENTA to minimize scan duration and potential hazard for patients. First, anatomical, cine, T2-weighted, rest-perfusion and LGE-CMR imaging were performed and assessed during the scan. If a CMR diagnosis was clear, scanning was stopped. When findings were normal or equivocal, adenosine stress-perfusion imaging was subsequently performed.

### Image acquisition

Patients were scanned on a 3T MR scanner (Tx series, Achieva, Philips Healthcare, Best, The Netherlands) equipped with maximum field gradients of 40 and 80 mT/m and slew rates of 100 and 200 mT/m/ms using a dedicated 32 element cardiac phased-array coil. The CMR protocol has been described previously [10] and included: (1) cine images in horizontal and vertical long axis, left ventricular (LV) outflow tract and multi-slice short axis fully covering the LV using a balanced steady-state free precession sequence for functional analysis, (2) multi-slice black blood T2-weighted imaging in short axis fully covering the LV using a double inversion recovery, turbo spin echo sequence for edema imaging, (3) first-pass perfusion imaging at basal, midventricular and apical level using a T1-weighted turbo gradient echo sequence during an intravenous bolus of 0.05 mmol/kg gadolinium (Gadovist®, Gadobutrol, Bayer Healthcare, Berlin, Germany) followed by a saline bolus flush (4 ml/sec), (4) LGE in the same imaging plane as was used for cine imaging, 10–20 min after an additional intravenous bolus of 0.1 mmol/kg gadolinium using a 2D T1-weighted gradient echo inversion recovery sequence to image scar, and (5) first-pass, adenosine vasodilator stress-perfusion imaging (after 140 µg/kg/min adenosine for 3 min) using the identical protocol as the rest-perfusion, ~ 30 min after rest-perfusion imaging.

### CMR analysis

CMR data were analyzed independently from the original assessment, blinded from the ICA and clinical data by two experienced observers and read in consensus (YvC and SB). Cine images were assessed for regional wall-motion abnormalities, T2-weighted and LGE images for the visual presence of edema and scar, respectively and rest- and stress-perfusion images for the presence of ischemia. Rest-perfusion imaging was only used to ascertain the stress perfusion defect. Each sequence was scored according to the American Heart Association 17-segment model [11], the true apex was excluded for the perfusion images.

The analysis comprised two steps. In step 1, cine, T2-weighted and LGE images were analyzed together. Underlying obstructive CAD (i.e. coronary etiology) was diagnosed in case of (1) regional wall-motion abnormalities and/or subendocardial/transmural hyperintensity on T2-weighted and/or LGE images in ≥ 2 segments or (2) at least 2 of the 3 sequences abnormal (i.e., regional wall-motion abnormalities, subendocardial/transmural hyperintensity on T2-weighted or LGE images) in a single segment (see Table 2). In case step 1 did not reveal a diagnosis, patients underwent stress-perfusion imaging in step 2. Underlying obstructive CAD was diagnosed when a regional, persistent perfusion defect was present in ≥ 2 adjacent segments, which was not considered an artifact [11]. The final analysis included the results of step 1 and 2 and was positive when either the combination of cine, T2-weighted imaging or LGE (step 1) or stress-perfusion imaging (step 2) suggested underlying obstructive CAD.

### ICA analysis

All ICA images were analyzed by an experienced interventional cardiologist (RT) blinded for the CMR and clinical data. The presence and severity of stenosis (absent, wall irregularities (< 20% diameter stenosis), 20–49% stenosis, 50–69% stenosis, 70–89% stenosis, 90–99% stenosis and total coronary occlusion) were scored for each segment. Obstructive CAD was defined as luminal diameter narrowing of ≥ 70% in any epicardial coronary artery.

### Adjudicated diagnosis committee

An independent committee (consisting of one interventional, one clinical and one imaging cardiologist) reviewed all available clinical data, including the CMR and ICA results. The following adjudicated diagnoses were made: ACS (either MI or unstable angina pectoris (UAP)), non-coronary but cardiac etiology (e.g., myocarditis, cardiomyopathy), or unknown etiology. The diagnosis of MI was based on the third universal definition [12].

### Statistical analysis

Continuous variables are presented as mean ± standard deviation when normally distributed (based on Shapiro–Wilk test and visual assessment of histograms) or as median and interquartile ranges for skewed data. Categorical variables are given as frequencies and percentages. To compare the groups with and without obstructive CAD the independent-samples t-test or Mann–Whitney U-test was used for normal and skewed continuous data, respectively. The Pearson’s chi-squared test (or Fisher’s exact test in case of small numbers) was used for categorical variables. To assess the diagnostic accuracy of CMR, sensitivity, specificity and overall accuracy were calculated to detect obstructive CAD (≥ 70% stenosis by ICA) and ACS. Analyses were performed on a per-patient level. All statistical analyses were carried out using SPSS (version 25.0, Statistical Package for the Social Sciences, International Business Machines, Inc., Armonk, New York, USA). A p-value < 0.05 was considered statistically significant.

## Results

### Study population

**Fig. 1:**
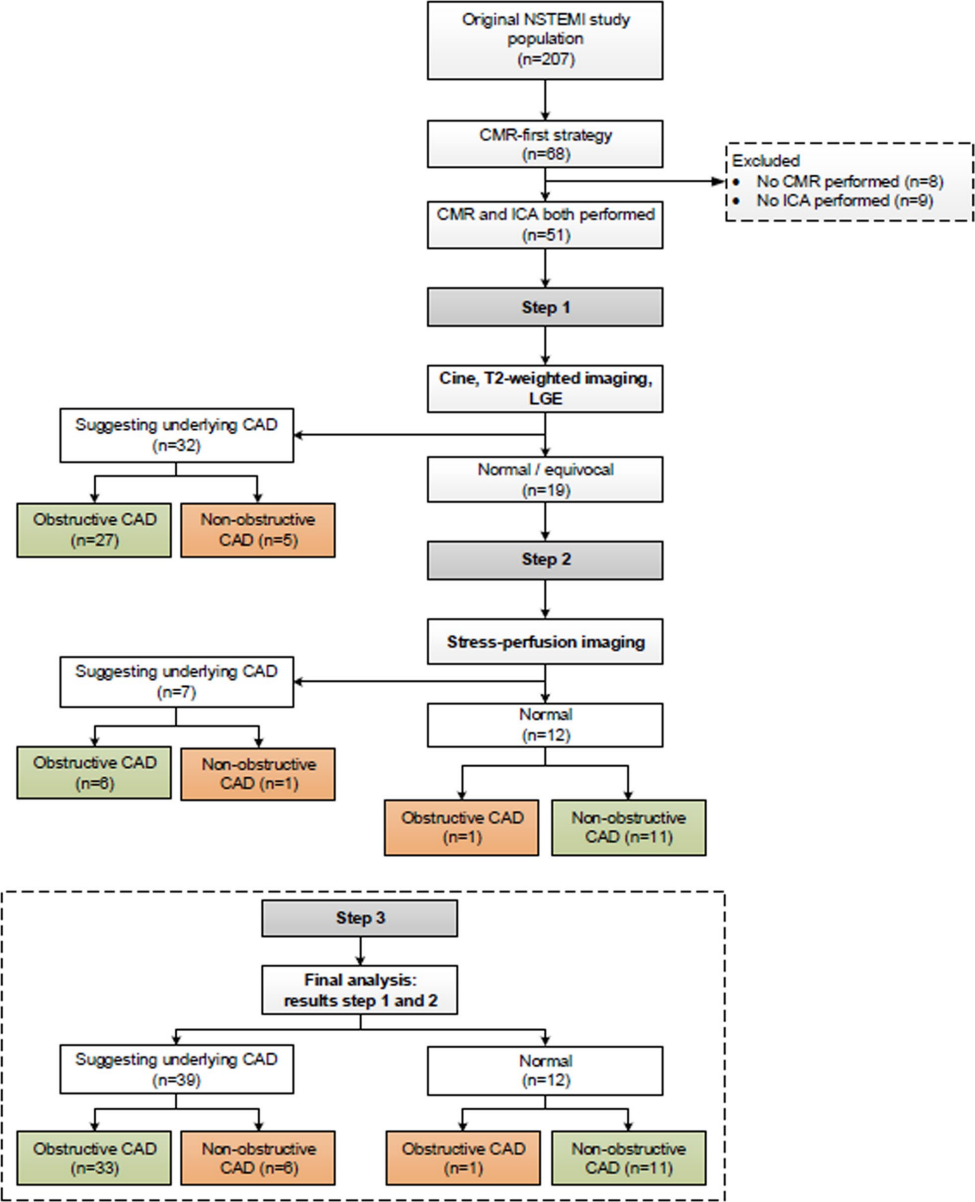
Patient flowchart. A stepwise analysis was used to investigate the diagnostic accuracy of cardiovascular magnetic resonance (CMR) to detect CAD (≥ 70% stenosis in any epicardial coronary artery). Green boxes: correct positive or negative results; orange boxes: false-positive or -negative results. Abbreviations: *CAD* coronary artery disease; *LGE* late gadolinium enhancement; *ICA* invasive coronary angiography; *NSTEMI* non-ST elevation myocardial infarction

The CMR-first arm of the CARMENTA trial comprised 68 patients. For this sub-analysis, 17 patients were excluded because either CMR (n = 8) or ICA were not performed (n = 9). Therefore, this sub-analysis is based on 51 patients (Fig. 1, Table 1).

**Table 1:** Baseline characteristics at initial presentation

	Total population (n = 51)	No-obstructive CAD (n = 17)	Obstructive CAD (n = 34)	P-value
Age (years)	63.1 ± 9.9	63.4 ± 9.5	63.0 ± 10.2	0.914
Male	26 (51)	5 (29)	21 (62)	**0.029**
BMI (kg/m^2^)	27.4 ± 4.2	28.4 ± 4.3	26.8 ± 4.1	0.198
*Concomitant vascular disease*				
Stroke/TIA	2 (4)	0 (0)	2 (6)	0.547
Peripheral vascular disease	4 (8)	1 (6)	3 (9)	1.000
*Cardiovascular risk factors*				
Hypertension	21 (41)	6 (35)	15 (44)	0.546
Diabetes mellitus	5 (10)	0 (0)	5 (15)	0.156
Hypercholesterolemia	15 (29)	4 (24)	11 (32)	0.746
Positive family history	27 (53)	9 (53)	18 (53)	1.000
Smoking	21 (41)	8 (47)	13 (38)	0.546
Number of CAD risk factors	1.8 ± 1.2	1.6 ± 1.0	1.8 ± 1.3	0.508
GRACE risk score	114 ± 22	115 ± 18	113 ± 24	0.801
*Physical examination*				
Heart rate (bpm)	71 ± 15	72 ± 17	71 ± 14	0.808
Systolic BP (mmHg)	148 ± 22	141 ± 15	152 ± 24	0.096
Diastolic BP (mmHg)	81 ± 13	79 ± 14	83 ± 13	0.332
*Laboratory assessment*				
Hemoglobin (mmol/L)	8.8 ± 0.7	8.9 ± 0.7	8.8 ± 0.8	0.894
Creatinin (µmol/L)	78 ± 15	76 ± 14	79 ± 16	0.537
Baseline hs-cTnT (ng/L)	53 (22–107)	48 (21–136)	56 (22–108)	0.865
Second hs-cTnT (ng/L)	91 (34–212)	47 (22–207)	104 (42–213)	0.294
CK (U/L)	148 (98–213)	148 (100–210)	146 (91–216)	0.646
CRP (mg/L)	2 (< 1–5)	4 (< 1–5)	1 (< 1–4)	0.095

Continuous data are presented as mean ± standard deviations or median (interquartile ranges), categorical data as frequencies (%). Significant P-values < 0.05 are shown in bold

Bold indicates significant value (P < 0.05)

*BMI* body mass index, *BP* blood pressure, *CABG* coronary artery bypass grafting, *CAD* coronary artery disease (defined as stenosis ≥ 70%), *CK* creatinine kinase, *CRP* C-reactive protein, *hs-cTnT* high-sensitivity cardiac troponin T, *TIA* transient ischemic attack

Two-thirds of patients (n = 34) had obstructive CAD, the majority (71%) having single-vessel disease. The average age was 63 ± 10 years. There were significantly fewer men in the non-obstructive CAD group compared to the obstructive CAD group (29% vs. 62%, P = 0.029). All remaining baseline characteristics were comparable between both subgroups (Tables 1 and 2). Most patients (90%) were at low-to-intermediate risk for 6-month death or MI with a GRACE risk-score of 114 ± 22 and were representative for the total CARMENTA population [9]. The median time between CMR and ICA was 1 day [1–3 days].

**Table 2:** Baseline imaging characteristics

	Total population (n = 51)	Non-obstructive CAD (n = 17)	Obstructive CAD (n = 34)	P-value
*Invasive coronary angiography*				
Non-obstructive CAD (< 70% stenosis)	17 (33)	17 (100)	0 (0)	
Single-vessel disease	24 (47)	0 (0)	24 (71)	
Multivessel disease	10 (20)	0 (0)	10 (29)	
*Cardiac magnetic resonance imaging*				
LVEDV (ml)	157 ± 40	148 ± 39	162 ± 40	0.245
LVESV (ml)	65 ± 24	62 ± 20	67 ± 26	0.502
LVSV (ml)	92 ± 22	86 ± 25	95 ± 20	0.173
LVEF (%)	59 ± 7	58 ± 8	60 ± 7	0.542
LV mass (gr)	107 ± 33	96 ± 28	113 ± 34	0.081
Suggesting obstructive CAD	38 (75)	6 (29)	33 (97)	** < 0.001**
LGE pattern				0.187
Infarction^a^	20 (39)	4 (24)	16 (47)	
Atypical^b^	7 (14)	2 (12)	5 (15)	
Absent	24 (47)	11 (64)	13 (38)	

Continuous data are presented as mean ± standard deviations, categorical data as frequencies (%). Significant P-values < 0.05 are shown in bold

*CAD* coronary artery disease (defined as ≥ 70% diameter stenosis in any epicardial coronary artery), *LGE* late gadolinium enhancement, *LV* left ventricular, *LVEDV* left ventricular end diastolic volume, *LVEF* left ventricular ejection fraction, *LVESV* left ventricular end systolic volume, *LVSV* left ventricular stroke volume

^a^Subendocardial or transmural LGE consistent with myocardial infarction

^b^Small focal areas of mostly mid-wall LGE not consistent with either myocardial infarction, myocarditis or non-ischemic cardiomyopathy

### Safety

In general, CMR scanning was well tolerated and no serious adverse events or complications occurred. Mild side effects included dyspnea (n = 1), nausea (n = 2) and recognizable chest pain (n = 2), that disappeared after discontinuation of adenosine. No scan had to be terminated prematurely.

### Diagnostic accuracy of CMR to detect obstructive CAD

**Fig. 2:**
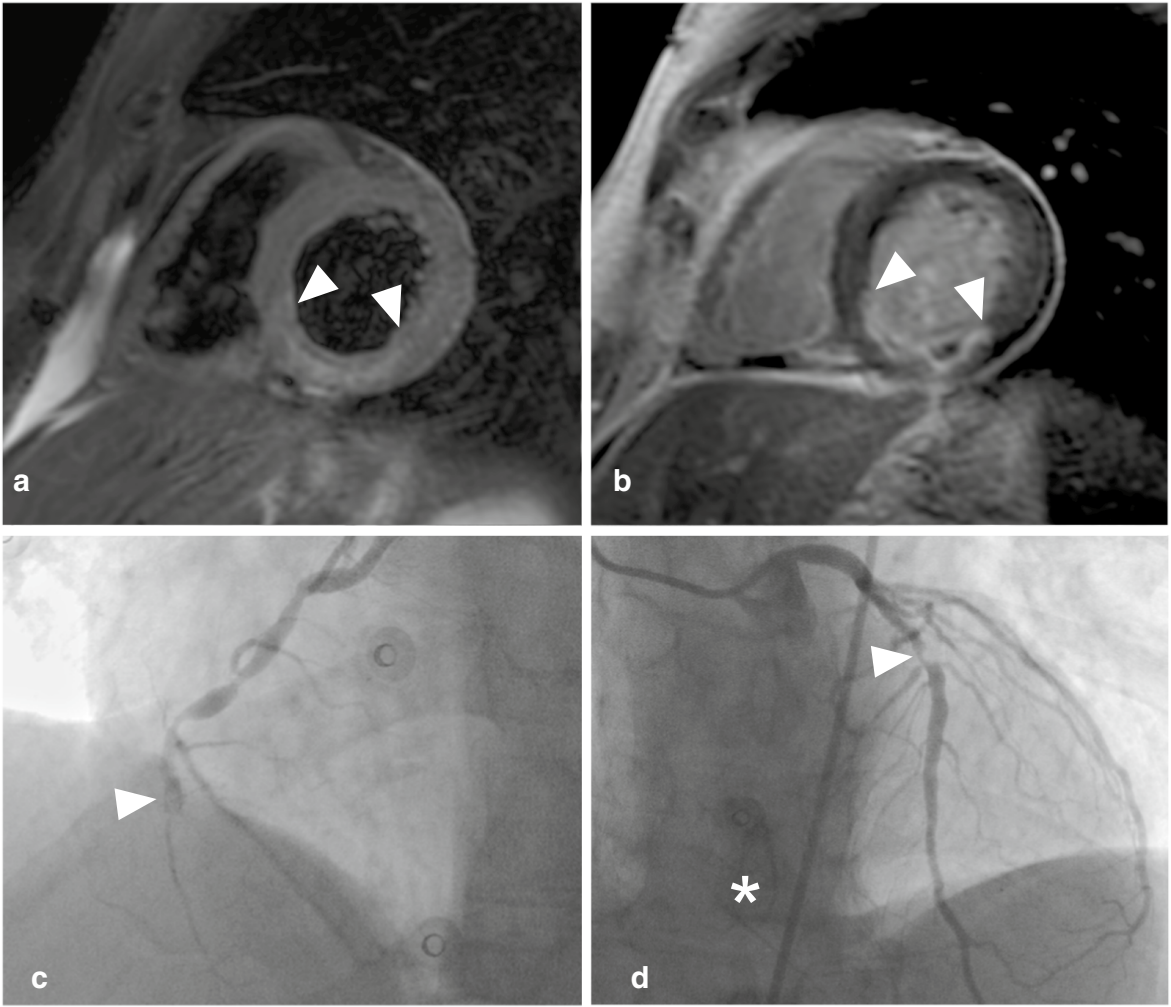
Patient example. Upper row: basal-inferior-inferoseptal edema on T2-weighted short axis left ventricular image (**a** arrowheads), 50–75% transmural basal-inferior-inferoseptal myocardial infarction with microvascular obstruction (**b** arrowheads). Lower row: total occlusion of the mid right coronary artery (**c** arrowhead), 71–90% mid left anterior descending artery stenosis (**d** arrowhead) with collaterals to right coronary artery (**d** asterisk)

**Fig. 3:**
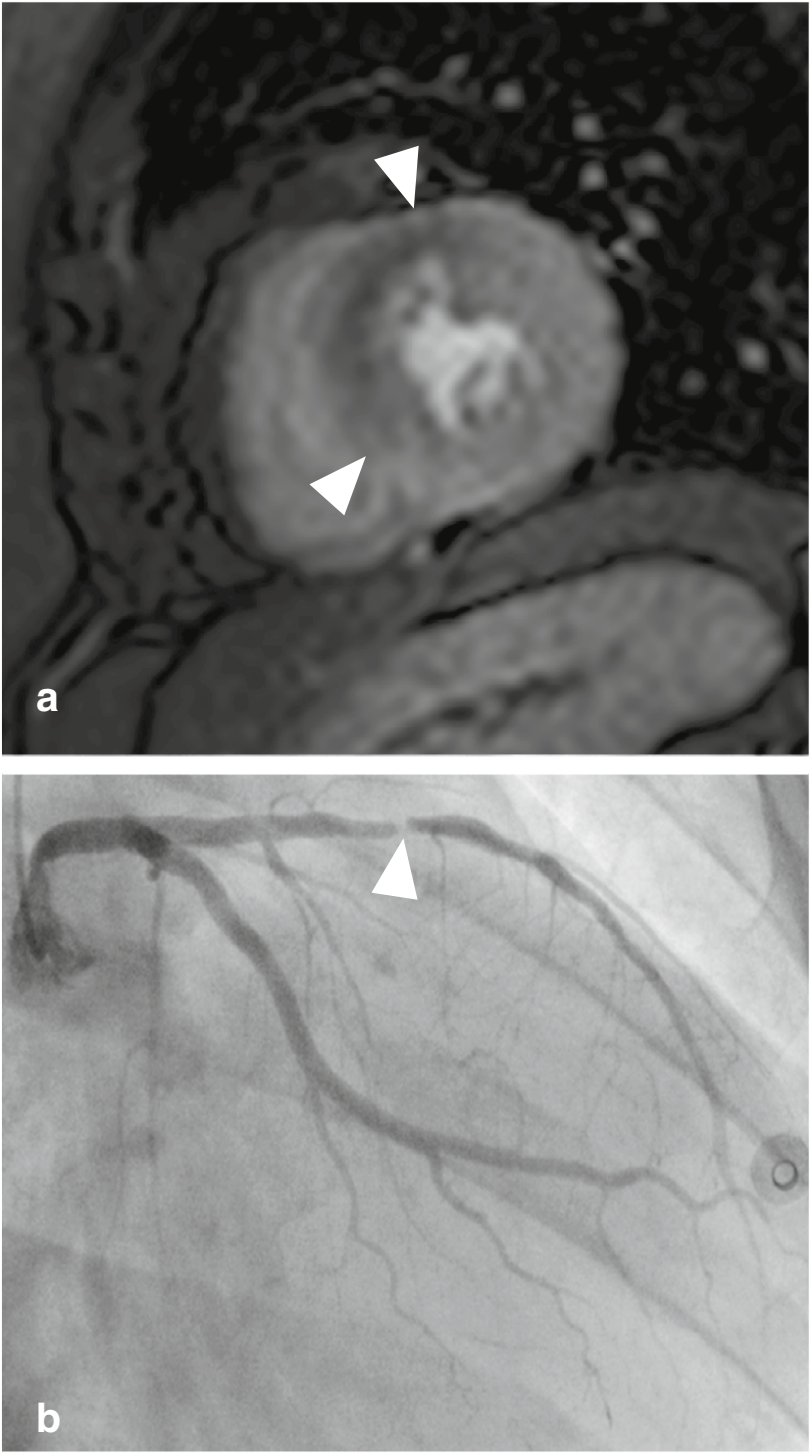
Patient example. **a**: adenosine stress-perfusion scan showing 50% transmural mid-anterior-anteroseptal perfusion defect (arrowheads). **b**: > 90% stenosis in the mid left anterior descending artery (arrowhead)

**Fig. 4:**
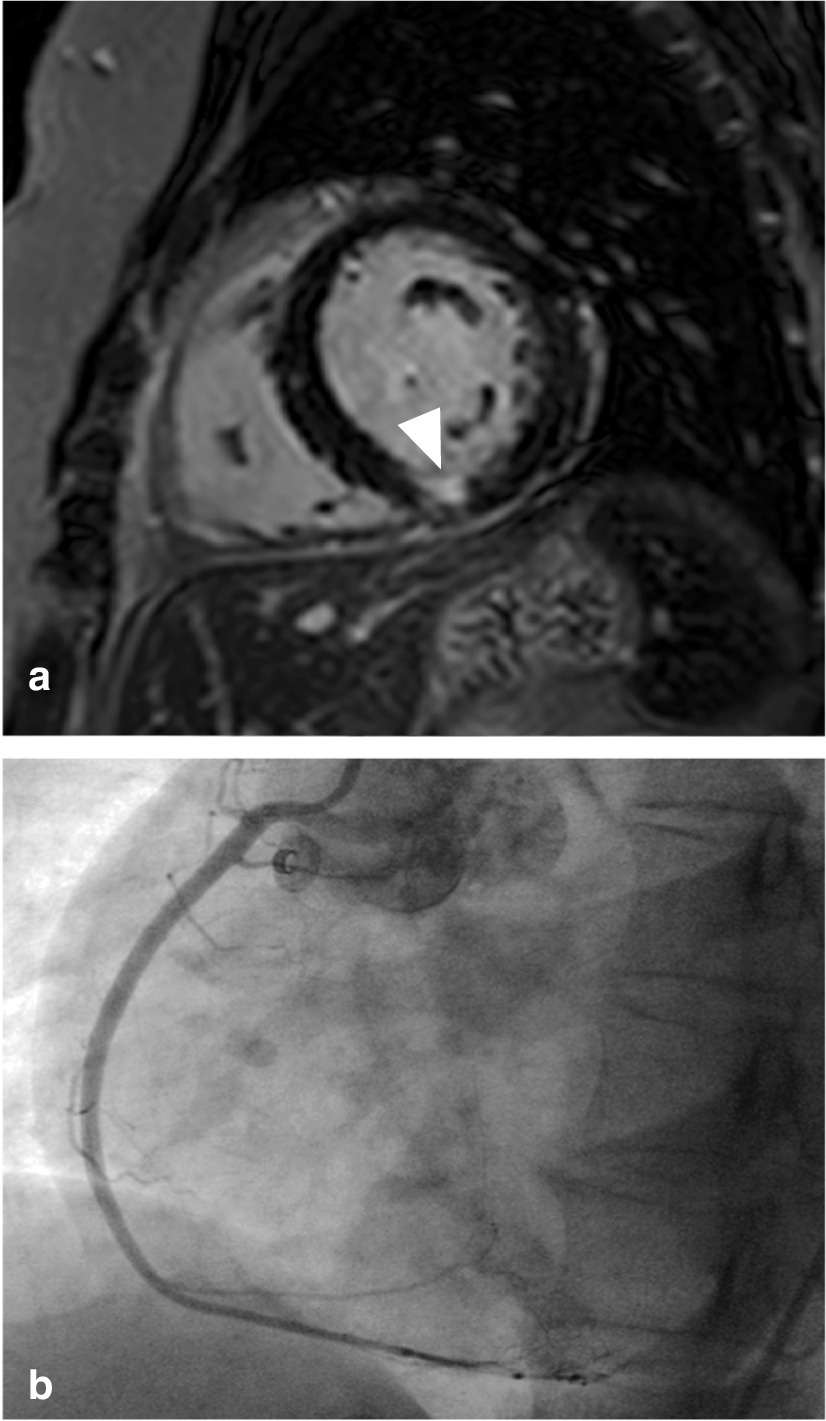
Patient example. **a**: limited subendocardial mid-inferior myocardial infarction (arrowhead). **b**: normal coronary angiogram, only right coronary artery shown

Combined cine, T2-weighted and LGE-CMR imaging (step 1) was evaluated in all 51 patients and suggested a coronary etiology in 32 (63%; Fig. 1). The sensitivity, specificity and overall accuracy to detect obstructive CAD were 79% (95%CI 62–91%), 71% (95%CI 44–90%) and 77% (95%CI 63–87%), respectively (Table 3). In 19 (37%) patients, the scan was either normal or equivocal and adenosine stress-perfusion imaging was added as a second step (Fig. 1). In this cohort, adenosine stress-perfusion imaging (step 2) had a sensitivity, specificity and overall accuracy of 86% (95%CI 42–100%), 92% (95%CI 62–100%) and 90% (95%CI 67–99%), respectively to detect obstructive CAD. When the results of both steps were combined, obstructive CAD was correctly identified in 33 of 34 patients (sensitivity 97% (95%CI 85–100%)). A coronary etiology was suggested by CMR in 6 out of the 17 patients (35%) with non-obstructive CAD, leading to a specificity of 65% (95%CI 38–86%). Overall accuracy remained high at 86% (95%CI 74–94%). After removing T2-weighted imaging from the initial combination of cine and LGE in step 1, followed by adenosine stress-perfusion imaging (step 2), sensitivity remained high at 94% (95%CI 80–99%), with a specificity of 65% (95%CI 38–86%) and accuracy of 84% (95%CI 71–93%). Patient examples are shown in Figs. 2, 3, 4.

**Table 3:** Diagnostic accuracy of CMR to detect obstructive CAD

	CMR sequence	Sensitivity (%)	Specificity (%)	Overall accuracy (%)
Step 1	Combination of cine, T2-weighted and LGE (n = 51)	79 (62–91)	71 (44–90)	77 (63–87)
Step 2	Vasodilator stress-perfusion imaging (n = 19)	86 (42–100)	92 (62–100)	90 (67–99)
Step 3	Final analysis (n = 51)	97 (85–100)	65 (38–86)	86 (74–94)

Numbers are presented as percentages (with 95% confidence intervals)

Obstructive CAD = coronary artery disease (defined as ≥ 70% stenosis in any epicardial coronary artery); LGE = late gadolinium enhancement. Step 1 abnormal: (1) regional wall-motion abnormalities, and/or subendocardial/transmural hyperintensity on T2-weighted and/or LGE in ≥ 2 segments or (2) at least 2 of 3 sequences abnormal in a single segment. Step 2 abnormal (only performed when step 1 was normal or equivocal): persistent subendocardial/transmural perfusion defect in ≥ 2 adjacent segments. Step 3 abnormal: abnormal results step 1 or 2

### Adjudicated final diagnosis and CMR findings

The adjudicated final diagnosis was ACS in 43 patients (84%; MI, n = 38; UAP, n = 5), stress cardiomyopathy (n = 1) and ‘unknown’ (n = 7).

All patients with obstructive CAD (n = 34) on ICA were finally diagnosed with ACS (MI, n = 31; UAP, n = 3). Of these, 33 (97%) had corresponding CMR abnormalities, while CMR was normal in one. In total, LGE imaging showed an infarct-pattern in 16 (47%), an atypical pattern (small focal areas of mid-wall LGE) pattern in 5 (15%) and was absent in 13 (38%) patients (Table 2).

In those with non-obstructive CAD (n = 17), 9 patients (53%) were finally diagnosed with ACS (MI, n = 7; UAP, n = 2). Of these 9 ACS patients, 4 patients (44%) showed an LGE pattern consistent with MI and 1 had a stress-induced perfusion defect in the absence of LGE. An atypical LGE pattern (atypical focal hyperenhancement in one segment) was observed in 2 patients and CMR was normal in 2 patients. Of the remaining 8 patients, 1 was diagnosed with a stress cardiomyopathy based on early CMR findings and follow-up imaging, while CMR was normal in 7 in whom the final diagnosis remained ‘unknown’. In total, LGE showed an infarct-pattern in 4 (24%), an atypical pattern in 2 (12%) and was absent in 11 (64%) patients (Table 2).

### Diagnostic accuracy of CMR to detect acute coronary syndrome as the final diagnosis

CMR correctly diagnosed 38 out of 43 (88%) patients with ACS. The sensitivity, specificity and overall accuracy of CMR to detect ACS were 88% (95%CI 75–96%), 88% (95%CI 47–100%), and 88% (95%CI 76–96%), respectively.

## Discussion

**Fig. 5:**
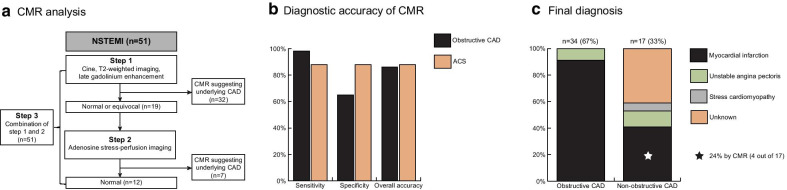
Graphical abstract. Panel **a**: stepwise CMR analysis, step 2 only performed in patients with normal or equivocal findings after step 1. Panel **b**: diagnostic accuracy of CMR to detect obstructive CAD (≥ 70% stenosis in any epicardial coronary artery) and acute coronary syndrome (ACS). Panel **c**: adjudicated diagnosis by independent committee, based on clinical data, CMR and invasive coronary angiography. Abbreviations: *CAD* coronary artery disease; *CMR* cardiovascular magnetic resonance imaging; *NSTEMI* non-ST elevation myocardial infarction

The present study shows that a comprehensive multicomponent CMR analysis integrating a stepwise combination of cine, T2-weighted and LGE with adenosine vasodilator stress-perfusion imaging has excellent sensitivity, reasonable specificity and overall good accuracy to detect obstructive CAD in patients with hs-cTnT positive suspected NSTEMI (Fig. 5a, b). Although one-third of patients had non-obstructive CAD, 24% nonetheless had an MI based on CMR (Fig. 5c). When using the full diagnostic potential of CMR and testing its accuracy to detect ACS rather than obstructive CAD, specificity increased from 65 to 88% while sensitivity remained high at 88% (Fig. 5b).

Early and accurate detection of acute MI is critical to initiate appropriate management and improve prognosis. This can be challenging, especially when high sensitivity troponins are (mildly) elevated and symptoms and/or the electrocardiogram less specific. In the appropriate clinical context and according to the fourth universal definition of MI, demonstrating either (regional) myocardial dysfunction or obstructive CAD can be diagnostic of acute MI [13]. In accordance with current guidelines, patients are often referred for ICA as initial diagnostic step to either demonstrate or exclude obstructive CAD [1]. However, 9–29% of patients with elevated troponin levels do not have obstructive CAD and are designated as having MINOCA [14, 15]. Our results and those of a ROMICAT-2 sub-study show that non-obstructive CAD is even more prevalent (33%) in the current hs-cTn era, increasingly posing clinicians for a diagnostic dilemma [16]. Because higher initial hs-cTn levels and/or larger delta values increase the likelihood of a coronary etiology (acute MI), this diagnostic uncertainty mainly arises in patients in whom hs-cTn values are only mildly elevated (so-called *observe zone*) [4]. Moreover, the introduction of hs-cTn assays into clinical practice has increased the referral of patients to ICA and associated revascularization procedures without improving outcome [17]. Altogether, this raises the question whether ICA is still the preferable initial diagnostic step for patients with acute chest pain, especially when hs-cTn levels are mildly elevated [1]. Non-invasive imaging techniques with similarly high sensitivity to detect obstructive CAD could be realistic alternatives to improve patient selection for follow-up invasive management.

Thus far, several non-invasive diagnostic strategies to evaluate acute chest pain and improve patient management in the emergency department and selection for ICA have been investigated. CTA allows accurate exclusion of CAD as well as identification of high-risk coronary plaque features that increase the likelihood of acute MI. Implementing CTA early in the diagnostic work-up of NSTEMI patients safely reduced the number of ICA compared to standard of care in one trial [9] but not in another [18]. Nonetheless, only ~ 40% of unselected patients in the emergency department are eligible for this approach because of contraindications for CTA or pre-existing CAD that may complicate interpretation of CTA findings during the index visit [4]. Furthermore, additional non-invasive testing is often needed to find a diagnosis when CAD has been excluded by CTA. CMR may be useful in this setting as it can accurately detect myocardial ischemia [19] and differentiate acute from chronic myocardial injury [20]. CMR also allows differentiating ischemic from other patterns of myocardial injury and is a key diagnostic tool in MINOCA, revealing a new diagnosis in 65–77% [5, 21].

Few studies have investigated the usefulness of comprehensive CMR imaging in patients with acute chest pain and suspected NSTEMI [22–28]. Although these studies cannot be compared directly because of differences in study population, design, prevalence of NSTEMI, CMR protocol and outcome measures, they all share a high sensitivity (up to 92%) and specificity (up to 96%). Our results corroborate that performing adenosine stress-perfusion CMR early (prior to ICA) is feasible and safe, because adenosine was well tolerated and no adverse events occurred [23, 24, 26, 28]. Our study is the first to investigate the diagnostic accuracy of early vasodilator stress-perfusion CMR in patients with NSTEMI with elevated hs-cTn levels. By itself, stress-perfusion CMR had good sensitivity and specificity (86% and 92%, respectively) to detect obstructive CAD when the combination of cine, T2-weighted and LGE-CMR was normal or equivocal. Adding stress-perfusion CMR to the model further increased the sensitivity from 79 to 97%, while minimally affecting specificity, which remained modest at 65%. This modest specificity is largely explained by the detection of MI by CMR in the absence of obstructive CAD. In agreement, the specificity of CMR to detect ACS was very high at 88%, suggesting that the use of ICA as reference standard should be reconsidered.

Indeed, one-third of patients (n = 17) did not have obstructive CAD. CMR led to a diagnosis in 6 (35%), of whom 4 (24%) still had an MI by CMR. Potential mechanisms may include coronary artery spasm or transitory thrombotic occlusion with self-lysis. Without CMR, these MI probably would have been missed and appropriate medical therapy withheld.

One patient was diagnosed with a stress cardiomyopathy, because of regional wall-motion abnormalities that normalized during follow-up and one patient with microvascular disease (regional hypoperfusion), who was finally diagnosed with unstable angina. The diagnostic yield of CMR in our study is lower than in previous reports, in which CMR led to a diagnosis in approximately two-thirds of patients with chest pain, elevated troponins and non-obstructive CAD. The prevalence of MI in our study was similar to these previous studies, but myocarditis was not observed at all in our study [5, 21]. The lower diagnostic yield in our study in comparison to previous studies, might be because patients had lower troponin levels and therefor too little necrosis to be detected by CMR. In addition, the requirement that both CMR and ICA had to be performed may have biased the results away from finding alternative diagnoses by CMR.

### Future perspectives

Our results strongly support the gatekeeping potential of early CMR in patients with suspected NSTEMI and select those who benefit most from additional ICA. When shown cost-effective, early CMR may become routine clinical practice in the future. The present work evaluated a range of sequences to establish the value of CMR. In this research setting, total duration of the CMR scan was approximately 1 h. Future work should investigate optimization of sequences to reduce total scan time and allow embedding in routine clinical practice. For example, our data already suggest that T2-weighted imaging may be omitted. A major advantage of early CMR is its overall diagnostic potential in a single investigation, leading to a diagnosis in a substantial number of patients, especially when obstructive CAD is excluded [21]. The diagnostic potential of CMR may be further increased when using myocardial blood flow quantification to improve the detection of microvascular disease and/or mapping techniques to improve the detection of myocarditis, iron overload, amyloid, Anderson-Fabry’s disease or other cardiomyopathies [29].

### Study limitations

This study is a sub-analysis of a larger randomized trial and included a relatively small number of patients. Nevertheless, this sample was representative of the original study population. While promising, prudence needs to be displayed to widely implement early CMR in all NSTEMI patients because our results may not apply to all patients and additional validation in multi-center studies is needed. In particular, patients with previously known CAD, known cardiomyopathy, evident type-2 MI and high-risk NSTEMI patients were excluded in the initial trial. For safety reasons, rest-perfusion imaging was performed prior to adenosine stress-perfusion imaging and this may have affected diagnostic accuracy. Although the sensitivity was still high at 86%, changing the order of performing stress prior to rest may have identified the only missed patient with obstructive CAD. Finally, we used an anatomical rather than a functional endpoint. Although functional assessment of CAD using fractional flow reserve (FFR) may overcome the limitations of luminography, usual care in NSTEMI is still based on visual interpretation of CAD severity. When we would have used FFR, this might have been to the advantage or disadvantage for CMR, since a visually obstructive lesion can be re-classified as non-significant or a non-obstructive lesion can be reclassified as significant by FFR. In the end, CMR has been shown to favorably compare with FFR [30].

## Conclusions

Non-obstructive CAD is prevalent in suspected NSTEMI and a comprehensive multicomponent CMR analysis including adenosine stress-perfusion is feasible, safe and a potentially useful gatekeeper for ICA. Incorporating CMR early in the diagnostic strategy can safely avoid ICA in a substantial number of NSTEMI patients.

## Data Availability

The dataset used and analyzed during the current study is available from the corresponding author on reasonable request.

## Abbreviations

ACS: Acute coronary syndrome
CAD: Coronary artery disease
CMR: Cardiovascular magnetic resonance
CTA: Computed tomography angiography
FFR: Fractional flow reserve
hs-cTn: High-sensitivity cardiac troponin
hs-cTnT: High-sensitivity cardiac troponin T
ICA: Invasive coronary angiography
LGE: Late gadolinium enhancement
LV: Left ventricle/left ventricular
LVEDV: Left ventricular end-diastolic volume
LVEF: Left ventricular ejection fraction
LVESV: Left ventricular end-systolic volume
LVSV: Left ventricular stroke volume
MI: Myocardial infarction
MINOCA: Myocardial infarction with non-obstructive coronary arteries
NSTEMI: Non-ST elevation myocardial infarction
UAP: Unstable angina pectoris
